# Multiomics tools for the diagnosis and treatment of rare neurological disease

**DOI:** 10.1007/s10545-018-0154-7

**Published:** 2018-03-13

**Authors:** L. M. Crowther, M. Poms, Barbara Plecko

**Affiliations:** 1 Division of Child Neurology, University Children’s Hospital Zurich, Zurich, Switzerland; 2 CRC Clinical Research Center, University Children’s Hospital Zurich, Zurich, Switzerland; 3 Radiz – Rare Disease Intiative Zurich, Clinical Research Priority Program for Rare Diseases, University of Zurich, Zurich, Switzerland; 4 Department of Pediatrics and Adolescent Medicine, Division of General Pediatrics, University Childrens’ Hospital Graz, Auenbruggerplatz 34/2, 8036 Graz, Austria

## Abstract

Conventional workup of rare neurological disease is frequently hampered by diagnostic delay or lack of diagnosis. While biomarkers have been established for many neurometabolic disorders, improved methods are required for diagnosis of previously unidentified or underreported causes of rare neurological disease. This would result in a higher diagnostic yield and increased patient numbers required for interventional studies. Recent studies using next-generation sequencing and metabolomics have led to identification of novel disease-causing genes and biomarkers. This combined approach can assist in overcoming challenges associated with analyzing and interpreting the large amount of data obtained from each technique. In particular, metabolomics can support the pathogenicity of sequence variants in genes encoding enzymes or transporters involved in metabolic pathways. Moreover, metabolomics can show the broader perturbation caused by inborn errors of metabolism and identify a metabolic fingerprint of metabolic disorders. As such, using "omics" has great potential to meet the current needs for improved diagnosis and elucidation of rare neurological disease.

## Introduction

The diagnosis of many childhood-onset neurological diseases is challenging due to often nonspecific clinical presentation or extreme rarity of the disease. It is estimated that ~6–8% of the general population is affected by a rare (orphan) disease, of which 80% are primary genetic and ~50% manifest in childhood. Delay or lack of diagnosis of rare diseases is common (Gahl et al. 2012), and the small number of patients diagnosed is a limiting factor for interventional studies, warranting improved diagnostic techniques.

Over the last 50 years, the development of a variety of techniques for analyzing biochemical compounds has enabled the discovery of various inborn errors of metabolism (IEM), including neurometabolic disorders that can affect the brain as part of a multiorgan manifestation. This is the case in intoxication-type disorders, in which circulating compounds (e.g., organic acids or ammonia) can damage various organs, including the brain, or in lysosomal storage disorders such as mucopolysaccharidosis type I (Hurler), or GM1 gangliosidosis, in which undegradable compounds accumulate in many tissues, including neuronal cells. Some IEM cause brain involvement exclusively, as seen in leukodystrophies (e.g., Canavan disease) or neurometabolic epilepsies (e.g., nonketotic hyperglycinemia). Biomarkers in cerebrospinal fluid (CSF), plasma, and dried blood spots (DBS) or urine have been identified for many of these disorders, enabling the development of newborn screening programs, selective screening, or specific diagnostic tests for a single disease. Still, detecting various compounds during diagnostic workup necessitates different assays, requires large sample volumes, and is laborious and time consuming. As well as helping in diagnosis of rare IEM, biomarkers are important surrogate parameters for treatment monitoring.

Recent developments in the fields of genomics and metabolomics offer enormous potential for elucidating genetic and metabolic causes of rare neurological disorders of hitherto unclear etiology. Combining these two powerful techniques enables discovery of novel biomarkers and support of pathogenicity—or lack—thereof, of variants of unknown significance in genes involved in metabolic pathways. Chromosomal microarray analysis and next-generation sequencing (NGS) are revealing an increasing number of disease-causing genes and mutations and are part of routine analysis in many specialized centers. Metabolomics is used to identify and quantify the small-molecule metabolic products (metabolome) in physiological or disease states. Untargeted metabolomics aims to analyze all measurable metabolites in a sample, while targeted metabolomics measures defined groups of chemically characterized and biochemically annotated metabolites (Roberts et al. 2012).

**Fig. 1:**
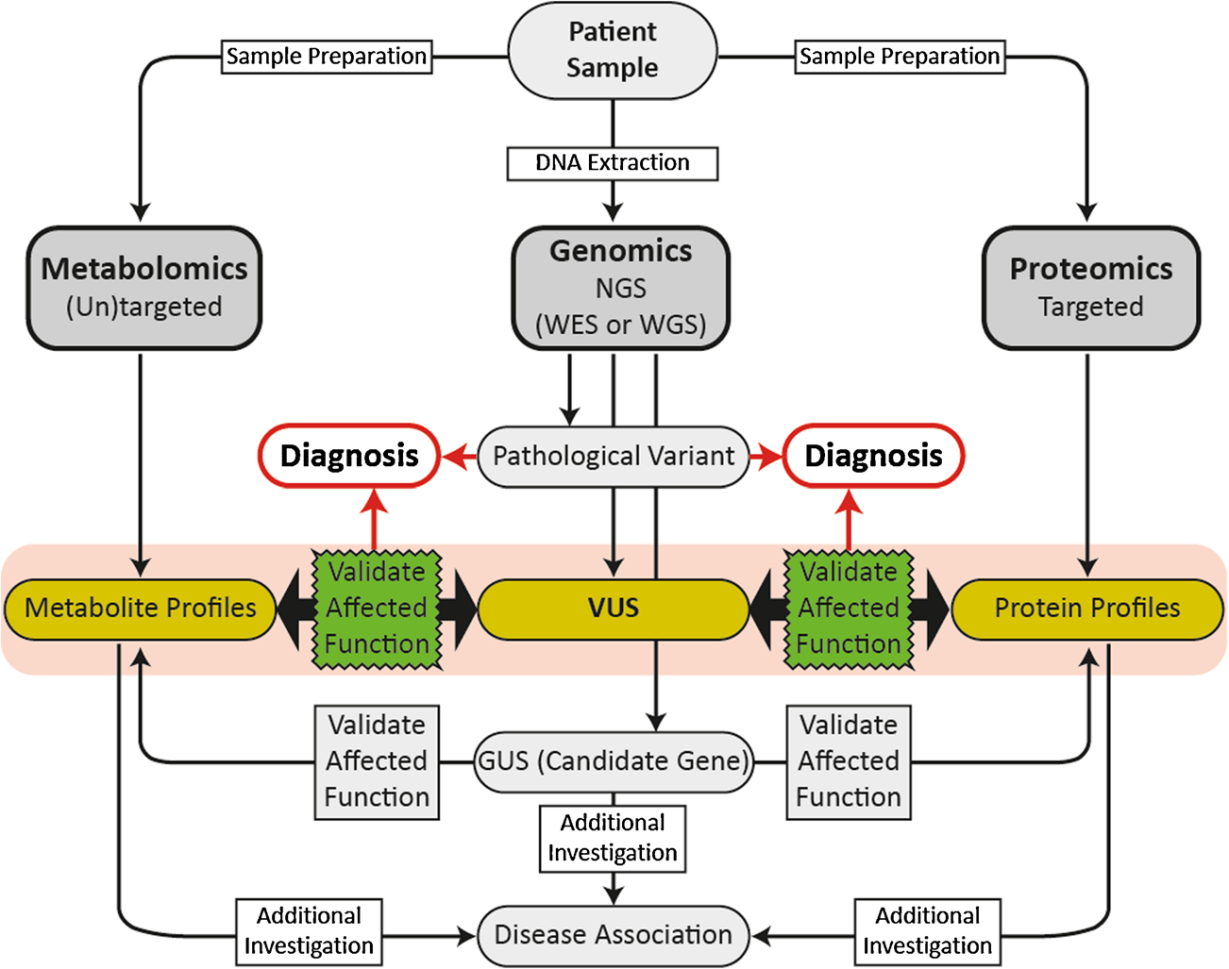
Workflow of combined omics’ approaches. Using combined “omics” techniques can be helpful for confirming effects of variants of unknown significance (VUS) on function of a disease gene or a gene of unknown significance (GUS) (candidate gene)

Recent collaborative multicenter studies have demonstrated the benefits of an “omics” approach in rare neurological diseases, incorporating the expertise of clinicians and experts in genomic and metabolomic analysis (Abela et al. 2016; 2017; Sirrs et al. 2015; Tarailo-Graovac et al. 2016). Analysis and interpretation of the large amounts of data obtained in such studies presents a challenge. In NGS, a high number of sequence variants need to be filtered and analyzed for pathogenicity and relevance to the disease. In metabolomics, the high number of features detected need biochemical identification to determine metabolites with alterations that might be disease related. Untargeted metabolomic profiling of patient samples has high sensitivity and does not only reflect endogenous biomarkers or disease-specific changes but is complicated by effects of diet, environment, therapeutics, and genetic background. This can confound interpretation and comparison of patient and control cohorts. However, when used in complement, metabolomics can aid in confirming pathogenicity of mutations identified by NGS. Conversely, identifying mutations in candidate genes from NGS can provide insights invaluable to the interpretation of metabolomic profiles (Fig. 1).

As such, a multiomics approach using metabolomic analysis used in combination with whole exome sequencing can be useful in interpreting the complex data obtained from each method. Here, we detail the benefits and challenges of the omics (genomics, proteomics, and metabolomics) and review published studies using multiomics in the field of rare neurological disease. Identifying proteomics biomarkers presents many challenges that are particularly difficult to overcome in studies on rare diseases. Most multiomics studies have therefore used a combined genomics–metabolomics approach, which we also focus on in this review.

## The omics

### Genomics

In the past decade, high-throughput sequencing has been made possible by advances in technology allowing faster and cheaper sequencing of large numbers of DNA sequences. This includes whole-genome sequencing (WGS) and whole exome sequencing (WES). Historically, Sanger sequencing was used to confirm suspected mutations in selected genes related to monogenic disease. This approach is less successful for diagnosing rare diseases with genetic heterogeneity, e.g., leukodystrophies with lack of typical magnetic resonance imaging (MRI) patterns), due to insufficient throughput and high costs for sequential sequencing of single genes (Neveling et al. 2013). Furthermore, NGS enables comprehensive analysis of the exome or genome for diagnosis and discovery of previously unrecognized disease-causing variants.

In brief, NGS involves amplifying and sequencing DNA fragments from samples to be analyzed using one of several available sequencing technologies (e.g., Illumina, Roche 254, Ion torrent, or SOLiD sequencing). In whole exome sequencing, only the protein-coding genes (exomes) are targeted and amplified, whereas for whole-genome sequencing, fragments from the entire genome are amplified. The millions of short-sequence reads generated are processed bioinformatically for alignment to a reference genome, and a list of variants (differences to the reference genome) is generated, which is then filtered to identify a subset of highly confident variants that are likely to be relevant to the disease. Variants in this subset are subsequently analyzed for significance and classified as belonging to one of five categories according to the American College of Medical Genetics (ACMG) guidelines (Richards et al. 2015):

- Pathogenic
- Likely pathogenic
- Likely benign
- Benign
- Variant of unknown clinical significance (VUS)

Classification of each variant takes into account available evidence indicating significance of the variant, including population-, computational-, functional-, and segregation data. In cases where the variant does not fall into either the pathogenic or benign category, it is classed as a VUS, and the effect of the variant on gene function needs to be further studied to determine likely pathogenicity.

The ACMG guidelines are intended to classify variants in genes with a definitive role in a Mendelian disorder. When no (likely) pathogenic variant is identified in any gene known to be associated with the disorder, variants in other genes that may be relevant can be considered as candidate genes (Fig. 1). Additional investigations are required to demonstrate affected function and provide evidence supporting the gene’s association with the disease before the variant can be considered pathogenic for the disease (Richards et al. 2015). As such, conventional assays, such as profiling of urine organic acids, plasma amino acids, acyl carnitines and neurotransmitters, or enzyme assays, can be useful and are necessary for assessing the functional effects of variants identified in NGS. Still, these techniques do not cover all metabolites and pathways that could be altered by hitherto unknown defects. In view of the 20,000 protein-encoding genes and that current text books list a total of only 530 monogenic IEM, it seems reasonable that by applying new techniques, many more IEM will be delineated in the near future.

Filtering and identifying which variants are disease causing presents the largest challenge in WGS and WES. Considering that there are 3–4 million variants and structural changes in a single individual when compared with the reference genome (Bentley et al. 2008; Johansson and Feuk 2012; Levy et al. 2007; Roach et al. 2010), and ~20,000–25,000 of those are in coding exons of genes (Wheeler et al. 2008; Ng et al. 2008; Ng et al. 2009), determining which identified variants cause disease is an enormous task and requires extensive time and expertise. The benefits of WES and WGS for diagnosing rare neurological diseases have been demonstrated in several recent studies comparing the use of NGS or conventional procedures as a first-line approach. In a group of 119 patients with neurodevelopmental disorders from 100 families, the use of WES and WGS reduced costs per family to a maximum of US $7640 from an average of US $19,100 of previously negative procedures, including laboratory tests, imaging procedures, electromyograms, and nerve-conduction-velocity studies. A molecular diagnosis was reached in 45% of patients with previously negative diagnostic testing, and time to diagnosis was estimated to accelerate by 77 months (Soden et al. 2014). Similarly, a recent analysis of the clinical utility of exome sequencing versus conventional genetic testing in selected patients with pediatric neurologic disorders revealed a diagnostic rate of 29.3% with NGS versus 7.3% with the standard-care pathway, >50% reduction in costs, and >80% reduction in time to diagnosis using the NGS approach (Vissers et al. 2017). In a group of patients with epileptic encephalopathy, use of targeted NGS panels increased the genetic diagnostic yield from <10% to >25% (Mercimek-Mahmutoglu et al. 2015). Similarly, diagnostic yields in the range of 22–25% have been reported with the use of epilepsy gene panels (Allen et al. 2016; Segal et al. 2016; Mercimek-Mahmutoglu et al. 2015).

While overall costs, time until diagnosis, and diagnostic yield indicate the usefulness of WES (or WGS) as a first-line diagnostic method in routine clinical diagnosis of suspected genetic neurological disease, limitations include availablility of resources required for analysis, interpretation of the complex data obtained, and further validation of VUS and candidate genes. Close collaboration between geneticists and clinicians is required for data interpretation with respect to deep clinical phenotyping and additional confirmatory investigations, including electroencephalogram (EEG), MRI, fundoscopy, etc. Furthermore, it is important that ethical issues regarding incidental findings and data ownership be considered carefully and regulated before establishing large-scale screening programs using these techniques.

### Proteomics

Proteomics can be described as the global identification and quantification of all proteins contained in a single biological sample. Despite recent improvements in instrumentation, experiment design, and data handling (Boersema et al. 2015), several challenges remain for the effective implementation of proteomics in clinical research. The excessively large dynamic range of protein concentrations in body fluids, especially blood, can result in a comparatively low positive identification rate for disease-specific proteins with intrinsically low concentrations (Beck et al. 2015). The most frequently measured enzymatic proteins are aspartate aminotransferase (AST) and alanine aminotransferase (ALT), mainly expressed in liver but also in muscle and kidney. These can be altered in various IEM affecting the liver, but their alteration is not sufficiently specific to meet criteria of a diagnostic biomarker. One example of an established protein biomarker for an IEM is ceruloplasmin in Wilson’s disease, which is always measured in combination with copper levels in serum and urine and is typically lowered as the respective transport protein.

Use of proteomics to identify biomarkers is limited due to substantial biological and experimental variability in clinical samples (even to a larger extent than in other omics approaches), which requires large patient and control cohorts, and means analysis is prohibitively expensive and time consuming (Drake et al. 2011; Schubert et al. 2017). Progress in the robustness and reproducibility of targeted experiments, however, makes proteomics a potentially valuable complementary technique for the detailed characterization of metabolic pathways (Crutchfield et al. 2016; Liu et al. 2013).

### Metabolomics

Metabolomics is the comprehensive analysis of the small-molecule metabolic products of a biological system. It has the potential to identify disease-related alterations in the metabolome and therefore can be used for biomarker discovery and confirmation of pathogenicity of mutations detected by NGS, if the relevant gene is involved in a metabolic pathway. As such, metabolomics is useful in discovery of new causes of neurological disease and further elucidation of already described IEM that hitherto lack a diagnostic marker.

**Fig. 2:**
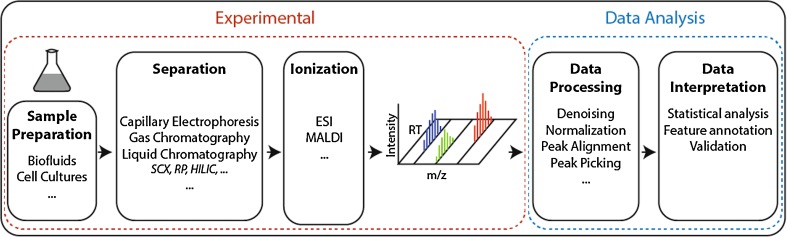
Workflow of a standard liquid chromatography/mass spectrometry (LC/MS) metabolomics experiment

The workflow of a metabolomics experiment can be divided into two consecutive sections (Smith et al. 2014): the experimental portion, which involves sampling, sample preparation, and measurement; and the bioinformatics portion, which includes data processing and interpretation (Fig. 2). Each step of metabolomics profiling needs to be carefully considered to obtain useful data from the complex output obtained. We now detail factors to be considered in each of these steps.

#### Sampling and sample preparation

Considering the high biological variation of metabolomic profiles in human biofluids due to effects of treatment, diet, environment, and genetic background, high numbers of age- and sex-matched patients and controls are needed, and it can be difficult to identify small-scale variations that can be relevant to the disease. These confounding factors can be more successfully controlled when using model systems such as cell culture or animal models, which may be useful first steps before undertaking more targeted analysis of human samples. Although CSF would be the ideal patient material for metabolomic analysis of rare neurological disease, it is difficult to obtain from sufficient numbers of patients and age and sex matched  healthy controls. Plasma is more easily obtained and can be useful in identifying novel biomarkers in central nervous system (CNS) disorders. One approach to minimizing variations due to diet is taking plasma samples after overnight fasting. However, as described by Hannelore (2013), overnight fasting can lead to increases in free fatty acids and branched-chain and aromatic amino acid concentrations, and these increases vary depending on the metabolic state of the patient and amount of glycogen stored in the liver.

Dried blood spots (DBS) offer advantages in terms of simple sample collection, storage, and transport, but they show limited metabolite stability (less than 1 week unless frozen), mitigating their advantages. So far their use is limited to the targeted analysis of metabolites or classes of metabolites with tested stability (Wilson 2011). Similarly, urine is easily obtainable, but there are conflicting reports on the stability of metabolites after urine collection (Dunn 2008; Gika et al. 2008; Lauridsen et al. 2007; Saude and Sykes 2007), and variable hydration status results in large differences in urine dilutions even from the same individual on different days (Ryan et al. 2011). Furthermore, urinary metabolite composition is heavily influenced by genetic and environmental factors, age, gender, and diet, which produce significant changes (Slupsky et al. 2007; Johnson and Gonzalez 2012; Bondia-Pons et al. 2013) and are likely to confound smaller, disease-specific effects, which limits the utility of urine for untargeted metabolomics approaches.

The use of highly sensitive analytical approaches in metabolomic experiments requires robust and reproducible sample preparation protocols and comparison to appropriate controls. The number of control samples required for a meaningful experiment can be estimated based on expected false discovery rates (Nyamundanda et al. 2013). It can be quite difficult to obtain sufficient numbers of healthy controls for analysis of human biofluids, as it is important that controls are age- and gender-matched to patients due to age- and gender-related variations in the metabolome. Collection of large cohorts of control samples can be time consuming. Ideally, all samples of interest and closely matched controls should be measured under the same conditions, preferably in one run. This can be managed in controlled studies but is a challenge in metabolomics for diagnostic testing of individual patients.

To minimize variation of the metabolome introduced during sampling, it is imperative that sampling, storage, and sample preparation is consistent. Current recommendations and precautions are outlined below.

#### Human biofluid sample handling

1. CSF
  To minimize metabolic activity of white blood cells (WBC) in CSF, which can affect the metabolome (Rosenling et al. 2009), it is recommended to centrifuge CSF samples immediately before snap freezing for storage at −80 °C. Even one freeze–thaw cycle can affect levels of transtherytin (Rosenling et al. 2009), while monoamine metabolites remain stable after repeated freeze–thaw cycles (Langlais et al. 1982). Therefore, such cycles should be minimized, and the effect on metabolites of interest should be monitored carefully.
2. Blood biofluids
  Preparation of serum requires incubation at room temperature for clotting prior to separation of serum, and clotting time affects the metabolomic profile (Timms et al. 2007; Teahan et al. 2006). Therefore, clotting time should be kept constant for metabolomics analysis. Preparation of plasma from whole blood does not require clotting, so samples can be placed on ice after collection. Metabolites are stabilized for up to 6 h when whole blood samples are stored at 4 °C prior to separation of plasma (Yin et al. 2013; Kamlage et al. 2014; Jobard et al. 2016). Plasma and serum sample storage at −80 °C is recommended, at which blood biofluids are stable for metabolomic analysis for at least 3 months (Jobard et al. 2016). It is recommended to minimize freeze–thaw cycles (Fliniaux et al. 2011; Teahan et al. 2006; Yin et al. 2015) as changes have been observed in several metabolites (Pinto et al. 2014; Yin et al. 2013; Fliniaux et al. 2011). It is also imperative that blood collection tubes used are consistent for all samples. The choice of blood collection tubes can affect the metabolome, and the best option depends on the analytical method used (Yin et al. 2015).

Plasma preparation includes a deproteinization step to precipitate high-molecular-weight species and subsequent removal by centrifugation, which results in loss of protein-bound metabolites. The sample is treated with an organic solvent/solvent mixture (typically methanol and/or acetonitrile), which is consequently removed through lyophilization or evaporation, and the sample is reconstituted in an appropriate solvent mixture for the desired analysis.

#### Cell culture sample preparation

Several factors can introduce variation in the metabolome during preparation of cell culture samples and should be minimized. Factors such as passage number should also be matched for comparisons to minimize potential effects on the metabolome due to time in culture. Trypsinization for removal of adherent cells from the culture dish is unsuitable for metabolomic profiling of cell culture extracts due to the prolonged medium-free incubation, which leads to an energy-depleted intracellular state;. Furthermore, damage to the cell membrane from trypsinization results in a variable portion of metabolites being released into the trypsin solution (Bordag et al. 2016; Martano et al. 2015; Quincy Teng et al. 2009).

The process of harvesting cultured cells for metabolomic analysis involves washing to remove contaminants from cell culture medium, quenching to stop intracellular enzymatic activities, and extraction of metabolites. Medium-free incubation time (during washing) should be minimized, and temperature changes before quenching should be avoided, as these can cause nonspecific variation in metabolite levels. A rapid water rinse prior to quenching improves liquid chromatography mass spectrometry (LC-MS) sensitivity and removes contaminants (Lorenz et al. 2011).

A protocol incorporating all these considerations has been optimized by Martano et al. (2015). Adherent cells are grown on glass coverslips, and during harvesting, plates are kept on a 37 °C heat block until coverslips are removed with forceps and washed quickly in a beaker of 37 °C H_2_0, then placed in cold solvent for quenching. Cells are scraped into the solvent and incubated 15 min on ice for extraction and subsequently frozen with liquid nitrogen. Samples are freeze -dried and re-extracted with an aqueous solution and mixed with a solvent appropriate for LC-MS analysis. A similar protocol was described by Bordag et al., but cells are grown on a Lumox® dish, and the membrane is cut with a scalpel to fall into warm washing buffer, followed by further washing, quenching, snap freezing, and extraction (Bordag N et al. 2016).

Differences in efficiency of cell transfer with cell scraping during harvesting can also introduce variation in total metabolite levels. To correlate metabolite levels with the number of cells in the harvested cell extracts, Muschet et al. reported a novel method for normalization based on correlating fluorescence-based DNA quantification with cell number using a small volume of the sample used for metabolomic profiling (Muschet et al. 2016). Other methods, such as measuring total protein concentration, cell count, or other methods of DNA quantification, also have a strong linear correlation with number of cells seeded (Silva et al. 2013). However, those require estimation of cell number from cells grown in parallel, rather than directly determining cell number from samples used for metabolomics, which is faster and more accurate.

#### Analytical methods

While consistent and reproducible sample preparation is imperative to minimize nonspecific variation in metabolite levels, the analytical method predetermines the class(es) of potentially observed metabolites. The two most common techniques are nuclear magnetic resonance (NMR) and ultra-high-performance LC–high-resolution mass spectrometry (UHPLC-MS). The Human Metabolome Database (Wishart et al. 2013) contains an ever-growing list of >74,000 unique metabolites (with a number of lipid variants in the order of 100,000) with an extensive range of different chemical and physical properties. Clearly, no single experimental setup can reliably measure all metabolites simultaneously.

NMR spectroscopy detects nuclei based on their magnetic properties, independent of chemical functionality, and represents a highly reproducible, relatively quick analytical method with the possibility of direct quantification over a large dynamic range (without the use of specific internal standards). It offers a considerable advantage in terms of unknown structure determination and can be used in tracing metabolic pathways when using substrates with stable isotope labelling. However, those advantages come at the price of comparably low sensitivity and resolution, effectively limiting the number of observable analytes and focussing on the most abundant ones. The constant development of novel techniques (Fan and Lane 2016) and the undeniable strength in identifying isotopomers still make NMR spectroscopy a viable technique for metabolomics, especially in combination with MS.

In most cases, MS-based metabolomic experiments include an additional initial separation step to distinguish signals from metabolites with the same mass but different structure. The most prevalent separation techniques are capillary electrophoresis (CE), gas chromatography (GC), and—most importantly—LC. It must be noted that every separation method has a limited dynamic range (e.g., strongly hydrophilic compounds cannot be sufficiently separated by a hydrophobic column and vice versa), making the choice of separation method one of the first selection criteria.

Another point of consideration for LC-MS experiments is effective ioniztion of analytes. For metabolomics, soft ionization methods, such as electron spray ionization (ESI) and matrix-assisted laser desorption/ionization (MALDI) are mostly used to preserve the entire molecule structure in contrast to harder ionization techniques, which would lead to fragmented compounds. In practice, only a relatively small percentage of molecules entering the ionization source are actually ionized, depending on their chemical composition and matrix. Accurate quantification of a compound hence often requires the use of a standard. Some classes of analytes, such as aldehydes and ketones, are also inherently difficult to ionize and therefore may require derivatization (Siegel et al. 2014; Miller et al. 2015). The various types of mass analyzers and detectors differ in resolution (the ability to differentiate between metabolites with similar masses), sensitivity (detection limit), and speed (some ionization methods require high-speed detectors, usually at the cost of resolution) and therefore differ in their spectrum of analytes detected.

Highly sensitive techniques, such as UHPLC-HRMS, are susceptible to minimal experimental and temporal changes in the system (column degradation, temperature, etc.), resulting in deviating measurements and potentially introducing systematic errors and artefacts. As this can severely hinder subsequent statistical analysis, it is recommended to analyze all samples in one experiment in randomized consecutive runs (Berg et al. 2013). Furthermore, authentic standards and/or reference samples (usually a pool of all samples) can be regularly dispersed throughout the experiment to control LC-MS stability and compare experiments from different runs (Sangster et al. 2006).

Experiment-driven, untargeted approaches are useful for studies with limited prior knowledge and exploration of previously uncharacterized conditions. They can be quite difficult to interpret due to the high number of detected features and potentially large differences in non-disease-related features (due to factors such as nutrition, medication, age, gender), which need to be identified and excluded from further analysis. Conversely, small changes that may be pathogenic can be missed quite easily with an untargeted approach. Targeted designs based on hypotheses of the disease mechanism focus on detecting single metabolites, metabolite classes, or specific pathways. They are generally less time consuming and often lead to clearer results.

#### Data processing and interpretation

Processing and analysis of experimental data represents the next important and challenging step in a metabolomics experiment. Raw data from LC-MS experiments usually contain thousands of features (peaks): mass-over-charge (m/z) intensities with a corresponding retention time (RT). Before data analysis, several processing steps, such as noise reduction and normalization and peak alignment and peak picking are required (Ejigu et al. 2013; Wu and Li 2016). Similarly, data generated from NMR measurements need to be processed (Fourier transformed), phased, and referenced before further analysis.

Well-established statistical tools, such as multivariate analysis, are subsequently applied to identify the most significantly altered peaks between two cohorts. Due to the large number of features detected, it is common practice to define a threshold for “meaningful” fold changes (Crews et al. 2009). However, differences in the metabolome should always be considered from a biological perspective. Concentration ranges of metabolites can vary significantly in their natural abundance, and the largest changes do not necessarily signify the most biologically relevant ones, whereas relatively small but consistent (significant) alterations can have a considerable biological effect (Crews et al. 2009). Setting thresholds that are too rigid can exclude small yet significant changes in the metabolome, whereas loosely defined cutoffs can result in data that is too complex to interpret. Choosing an appropriate fold-change threshold must be carefully considered.

Correlating significant features to specific biologically relevant metabolites (feature annotation) remains the most important and challenging step in a metabolomics experiment. As each mass (or NMR peak) correlates with multiple possible assignments, obtaining correct assignments is inherently difficult. Even the use of multidimensional MS and NMR experiments, which additionally provide structural information for the most abundant features, has only led to incremental improvement in this area. Traditionally, features have been compared with established databases, with the goal of assigning as many features as possible. Even though metabolite databases are constantly expanding, coverage of the metabolome is often still insufficient for global metabolomic experiments. In recent years, several assignment approaches have been developed with the aim of incorporating pathway and network information in the annotation process (Li et al. 2013; Uppal et al. 2017; Grapov et al. 2015; Karp et al. 2002) by initially identifying relevant metabolite pathways and networks (thus limiting the number of possible positive matches) before the traditional step of feature assignment. Developments in the relatively young field of data mining, and constant improvement in annotation algorithms, represent considerable advances in feature annotation, as is indicated by growing numbers of participants in the critical Assessment of Small Molecule Identification (CASMI) contest (Schymanski et al. 2017). However, feature annotation from large data sets remains a time consuming and extremely challenging step necessary for meaningful interpretation of metabolomic experiments.

## Multiomic studies in rare neurological disease

Several studies, including ours, have used a “multiomics” approach using NGS in patients with undiagnosed neurological disease and untargeted or targeted metabolomic profiling to examine the effects of mutations on the metabolome (Abela et al. 2016; 2017; Sirrs et al. 2015; Tarailo-Graovac et al. 2016). This multidisciplinary and often multicentric approach has enabled elucidation of etiology in several cases of undiagnosed rare neurological disease and led to identification of  novel potential biomarkers or metabolic profiles with potential diagnostic utility. Furthermore, identification of potentially disease-causing mutations and subsequent metabolomic analysis of patient samples, patient fibroblasts, or mouse models has demonstrated metabolic perturbations relevant to identified mutations in candidate genes and provided evidence of disease causality (Ait-El-Mkadem et al. 2017; Ouyang et al. 2016).

### Biomarker identification

In combination with whole exome sequencing of patient cohorts, metabolomics has been used to identify potential biomarkers or metabolomic fingerprints which could be useful for diagnostics. To be successfully used in a clinical setting, biomarkers need to be disease-specific, sensitive to pathological changes and highly reproducible. Potential biomarkers identified in metabolomic studies subsequently require confirmation and validation through standard analytical methods before being used in clinical routine. Biomarkers can then be used in multiple different clinical applications, from screening and diagnosis to prognosis and prediction as well as monitoring.

From a cohort of patients with epileptic encephalopathy, we identified two patients with mutations in the spermine synthase (*SMS*) gene, causing Snyder–Robinson syndrome (SRS) (Abela et al. 2016). Spermine synthase converts spermidine to spermine, and previously diagnosis of SRS was performed by sequencing the *SMS* gene and/or determining the spermine/spermidine ratio in lymphoblasts (Sowell et al. 2011). In plasma from three patients from two families, we detected elevated spermidine and identified elevated levels of N8-acetylspermidine and isoputreanine, which are derived from spermidine and putrescine. As such, N8-acetylspermidine was identified as a novel potential plasma biomarker for SRS.

We also identified a metabolic profile for aconitase deficiency, which is potentially useful for diagnosis (Abela et al. 2017). After identifying a patient with mutations in *ACO2* from the epileptic encephalopathy cohort described above, we examined the metabolomic profile of five Aco2-deficient patients from four families and identified a disease-specific metabolic fingerprint of decreased isocitrate, cis-aconitate, and α-ketoglutarate, with unchanged succinate, fumarate, and malate levels, in plasma. This study also demonstrates the utility of metabolomic analysis in examining the effect of mutations on enzyme function, demonstrating that the function of Aco2 was affected by the variants detected by whole exome sequencing.

### Confirmation of disease causality of mutations and treatment indications

Apart from use of a multiomics approach for biomarker discovery, multiomic studies have also provided evidence supporting disease causality of mutations identified in patients with rare neurological disease, elucidated pathogenicity, and in some cases indications for potential treatment strategies. In a recent study by Ait-El-Mkadem et al., sequence variants in *MDH2* were identified by WES in three unrelated patients with early-onset mitochondrial phenotype with generalized hypotonia, psychomotor delay, and refractory epilepsy (Ait-El-Mkadem et al. 2017). Metabolomic analysis of fibroblasts from one patient demonstrated accumulation of the substrate of MDH, malate, and its precursor in the Krebs cycle—fumarate. Elevated malate and fumarate was also detected in plasma from one patient. As such, metabolomic analysis identified metabolic perturbations associated with the variant in *MDH2*.

Ouyang et al. identified mutations in *GPT2* in patients with intellectual and developmental disability from two large consanguineous kindreds by WES and then studied the effect of *GPT2* deficiency in mice using targeted MS-based metabolomics and metabolite-set enrichment analysis (Ouyang et al. 2016). *GPT2* encodes for glutamate pyruvate transaminase 2, which catalyzes the reversible addition of an amino group from glutamate to pyruvate, yielding alanine and α-ketoglutarate. Metabolomic analysis demonstrated abnormal profiles involving the tricarboxylic acid (TCA) cycle (alanine, citrate, isocitrate, succinate, fumarate, and malate) and neuroprotective mechanisms [glutathione, glutathione disulfide, cysteine, nicotinamide adenine dinucleotide phosphate, reduced (NADPH), nicotinamide adenine dinucleotide phosphate (NADP+), cystathionine, and folate] in *Gpt2*-null mice. A loss of function mutation in *GPT2* had previously been identified in three siblings with intellectual disability from one family, and apart from low plasma alanine, all amino acid levels were normal (Celis et al. 2015). While the mouse studies demonstrated effects of *Gpt2* deficiency on brain growth during postnatal development, the different amino acid profiles in mice and humans demonstrate that effects seen in mice do not necessarily occur in humans (Celis et al. 2015; Ouyang et al. 2016).

In a study by Tarailo-Graovac et al. (2016), a multidisciplinary approach was used to identify and characterize causal variants in patients with intellectual development disorder and unexplained metabolic phenotype. NGS on samples from 47 patients identified disease-causing or potentially disease-causing mutations in 68% of patients. In several of these cases, targeted metabolomics played a role in confirming pathogenicity of mutations detected by WES and identifying new causes of IEM, such as variants in *FAA2H* or *NANS*.

In one patient in the same study with autistic features before the age of 2 years, a rare missense mutation was detected by WES in *FAAH2* encoding fatty-acid amide hydrolase 2 (FAAH2), which has a role in lipid metabolism and mediates degradation of endocannabinoids but had not been linked to neurological disorders. An abnormal whole-blood acylcarnitine profile was seen, with ten-fold elevations in medium-chain species, and targeted quantitative lipidomics showed perturbations in multiple lipid species in patient serum compared with ten controls, including elevations in many long-chain species (Tarailo-Graovac et al. 2016; Sirrs et al. 2015). Studies using fibroblasts from the patient demonstrated reduced FAAH2 activity and altered levels of endocannabinoid metabolites.

In another patient with epileptic encephalopathy and dysmorphic features, sequence variants were identified in the *NANS* gene (encoding for N-acetylneuraminic acid phosphate synthase) and correlated with increased levels of the NANS substrate N-acetylated mannosamine in urine, plasma, and CSF. Animal models of NANS deficiency were amenable to treatment with N-acetylneuraminic acid (Tarailo-Graovac et al. 2016). As such, WES and detection of metabolic perturbations elucidated pathogenicity and provided indications for possible treatment.

## Conclusions

Recent studies have demonstrated how the combined use of genomics with metabolomics can assist with the significant challenges each method faces in identifying pathological mutations and metabolic perturbations in rare neurological disease. This combined approach has the potential to meet the significant challenges of diagnosing rare neurological disease. Bioinformatic integration and analysis of raw data obtained from multiomics techniques (any combination of genomics, proteomics, and metabolomics) would represent a huge development with potential for large-scale studies and lead to significant advances in elucidating causes of rare disease and identifying biomarkers. However, as described in this review, genomics, proteomics, and metabolomics still require extensive and time-consuming human input for correct and reliable data interpretation. The omics technique gives categorically different outputs that are not linearly relatable (gene mutation, protein expression, metabolite concentration changes) and are therefore inherently difficult to compare directly and incorporated bioinformatically. A truly combined bioinformatically integrated omics approach is not yet feasible, and individual techniques are optimally used in a complementary manner.

The multiomics approach is increasingly providing knowledge and diagnostic tools, such as biomarkers, that are invaluable in rare diseases. The use of these methods is rapidly expanding, and technologies are continually being improved, indicating that the future for the omics, and its application in diagnosis and usefulness in studying rare diseases is bright.
